# A phase 2 randomized trial of safety and pharmacokinetics of IgPro20 and IgPro10 in patients with diffuse cutaneous systemic sclerosis

**DOI:** 10.1093/rheumatology/keaf066

**Published:** 2025-02-05

**Authors:** Christopher P Denton, Otylia Kowal-Bielecka, Susanna M Proudman, Marzena Olesińska, Margitta Worm, Nicoletta Del Papa, Marco Matucci-Cerinic, Jana Radewonuk, Jeanine Jochems, Adrian Panaite, Amgad Shebl, Anna Krupa, Yannick Allanore, Jutta H Hofmann, Maria J Gasior

**Affiliations:** Institute of Immunity & Transplantation, University College London, London, UK; Department of Rheumatology and Internal Medicine, Medical University of Bialystok, Bialystok, Poland; Rheumatology Unit, Royal Adelaide Hospital and Discipline of Medicine, University of Adelaide, Adelaide, Australia; Department of Connective Tissue Diseases, National Institute of Geriatrics, Rheumatology and Rehabilitation, Warsaw, Poland; Division of Allergy and Immunology, Department of Dermatology, Venerology and Allergology, Charité Universitätsmedizin, Berlin, Germany; Department of Rheumatology, Scleroderma Clinic, Clinica Reumatologica, ASST Gaetano Pini-CTO, Milan, Italy; IRCCS San Raffaele Hospital Cardiac Surgery Unit, UNIRAR and Division of Clinical and Experimental Rheumatology, IRCCS San Raffaele Hospital, Milan, Italy; University Vita Salute San Raffaele, Milan, Italy; Biostatistics, CSL Behring LLC, King of Prussia, PA, USA; Clinical Pharmacology, CSL Behring LLC, King of Prussia, PA, USA; Global Safety and Pharmacovigilance, CSL Behring AG, Bern, Switzerland; Clinical Safety and Pharmacovigilance, CSL Innovation GmbH, Marburg, Germany; Clinical Pharmacology, CSL Behring LLC, King of Prussia, PA, USA; Rheumatology Department, Hôpital Cochin, APHP, INSERM 1016, Paris, France; Clinical Development, Immunology, CSL Behring GmbH, Marburg, Germany; Clinical Development, Immunology, CSL Behring LLC, King of Prussia, PA, USA

**Keywords:** pharmacokinetics, subcutaneous immunoglobulin, intravenous immunoglobulin, systemic sclerosis

## Abstract

**Objectives:**

The primary objective was the safety of s.c. immunoglobulin, IgPro20 (Hizentra, CSL Behring) in adults with dcSSc. Secondary objectives included pharmacokinetics and relative bioavailability of IgPro20, and safety and pharmacokinetics of IVIG, IgPro10 (Privigen, CSL Behring).

**Methods:**

In this prospective, multicentre, randomized, open-label, crossover phase 2 study (NCT04137224), patients (aged ≥18 years) with dcSSc were assigned to 16 weeks of IgPro20 (0.5 g/kg/week) followed by 16 weeks of IgPro10 (2 g/kg/4 weeks over two to five sessions), or vice versa. Treatment-emergent adverse events (TEAEs), serious adverse events (SAEs), infusion site reactions (ISRs), clinical tests, pharmacokinetic and bioavailability were assessed.

**Results:**

Twenty-seven patients were randomized from 9 October 2019 to 31 August 2021. In total, 22 patients (81.5%) experienced 107 TEAEs (IgPro20, 49; IgPro10, 58); most were mild/moderate. Six patients (22.2%) experienced 10 SAEs (IgPro20, 6; IgPro10, 4); no treatment-related SAEs and no deaths were reported. IgPro20 ISR rate was low (2 per 100 infusions). Maximum IgG concentration [mean (s.d.)] was numerically lower following IgPro20 [23.7 (1.2) g/l] *vs* IgPro10 [46.1 (1.2) g/l], as was the geometric mean dose-normalized, baseline-corrected area under the concentration–time curve from time point 0 to tau [IgPro20, 44.8 (1.4) h*g/l; IgPro10, 60.2 (1.4) h*g/l]. The bioavailability of IgPro20 relative to IgPro10 was 76.1%.

**Conclusion:**

This study shows that in patients with dcSSc, safety, pharmacokinetic and bioavailability profiles of IgPro20, and safety and pharmacokinetics of IgPro10, are similar to those observed in other approved indications.

**Trial registration:**

ClinicalTrials.gov, https://clinicaltrials.gov, NCT04137224

Rheumatology key messages

- Safety, pharmacokinetics and bioavailability assessment begins clinical trial assessment of immunoglobulin therapy in dcSSc.
- S.c. immunoglobulin is well tolerated in adults with dcSSC, with acceptable safety, pharmacokinetic and bioavailability profiles.
- IgPro10/IgPro20 had acceptable pharmacokinetic and safety profiles to warrant further efficacy investigation in dcSSC.

## Introduction

SSc is a chronic autoimmune rare, immune-mediated connective tissue disorder, with a worldwide prevalence rate estimated at 17.6 per 100 000 [1–3]. The cumulative survival for patients 10 years post-diagnosis is 62.5%, making SSc one of the most life-threatening rheumatic diseases [4–6]. The disease is characterized by progressive vascular damage and organ fibrosis, with most patients presenting with skin thickening with variable internal organ involvement [2, 7, 8].

The two main subsets of SSc are defined by the distribution of skin thickening: lcSSc and dcSSc [8]. Patients with dcSSc (20–40% of SSc cases) generally experience early rapid progression of skin thickening and are at high risk for early, widespread and severe internal organ involvement [9], associated with increased morbidity, mortality and a poor quality of life [9, 10].

Current treatments of SSc are organ-based and primarily aimed at improving symptoms and managing complications [11]. Currently, systemic therapeutic options (primarily immunosuppressants) are used off-label, are only partially effective and are associated with adverse events (AEs), including severe infection [11, 12]. With no approved disease-modifying treatment for SSc, new therapeutic options are needed.

With the implication of immune dysregulation in SSc, IgG therapy might be beneficial for patients with SSc as it acts on various pathogenic mechanisms of autoimmune diseases [2, 13]. IgG therapy has been used as a treatment for autoimmune disorders for many decades and has the advantage of an excellent safety profile with no increased risk of infection, as it is not an immunosuppressant [13–16]. IgPro20 [20% s.c. human immunoglobulin (SCIG), Hizentra, CSL Behring] and IgPro10 [10% human IVIG, Privigen, CSL Behring] have existing approvals for autoimmune diseases and as immunoglobulin replacement therapy [17–20]. There is limited and inconclusive evidence on IVIG use in patients with SSc, including lcSSc and dcSSc, while some observational studies have indicated a benefit of IVIG for multiple clinical manifestations, a randomized controlled trial of IVIG in dcSSc did not meet its primary endpoint, although it did indicate that repeated IVIG may produce a stronger efficacy effect [16, 21–24]. No trials on the use of SCIG in patients with SSc have been published to date, except for one case published on the use of SCIG products in a patient with progressive SSc [25].

Experience in other indications shows that the pharmacokinetic (PK) and safety profiles of SCIG and IVIG are different, and mostly specific to the route of administration. SCIG results in higher trough, lower peak and reduced fluctuation of serum IgG levels than those observed with IVIG [26]. SCIG is also characterized by a lower rate of systemic AEs and a minimal ‘wearing off’ effect at the end of the dosing interval compared with IVIG [27, 28]. The most common AEs associated with SCIG products are local infusion site reactions (ISRs), which are predominantly mild, quickly resolve without specialized treatment and usually decrease over time [27]. Low rates of systemic AEs, flexible dosing regimens, good quality of life and cost savings are all important advantages of SCIG administration [26, 27, 29–31].

The safety of SCIG in SSc requires further evaluation as the change in skin and s.c. tissues in SSc leads to fibrosis, lymphatic vessel attenuation, vasculopathy and sometimes ulceration [2, 32, 33], which may impact the overall safety and PK (e.g. absorption) profile of IgG through s.c. administration. Therefore, before efficacy and safety of a SCIG product is assessed in a large-scale clinical study, a separate investigation of the safety, PK and relative bioavailability of SCIG in patients with dcSSc is warranted.

The primary objective of this prospective, multicentre, randomized, phase 2 study was the evaluation of the safety of SCIG (IgPro20) in adults with dcSSc by recording of AEs, treatment-emergent adverse events (TEAEs), AE of special interests (AESIs), ISRs and associated clinical tests. The secondary objectives were assessing PK and relative bioavailability of IgPro20, and the safety profile and PK of IgPro10.

## Methods

### Study design

**Figure 1.**
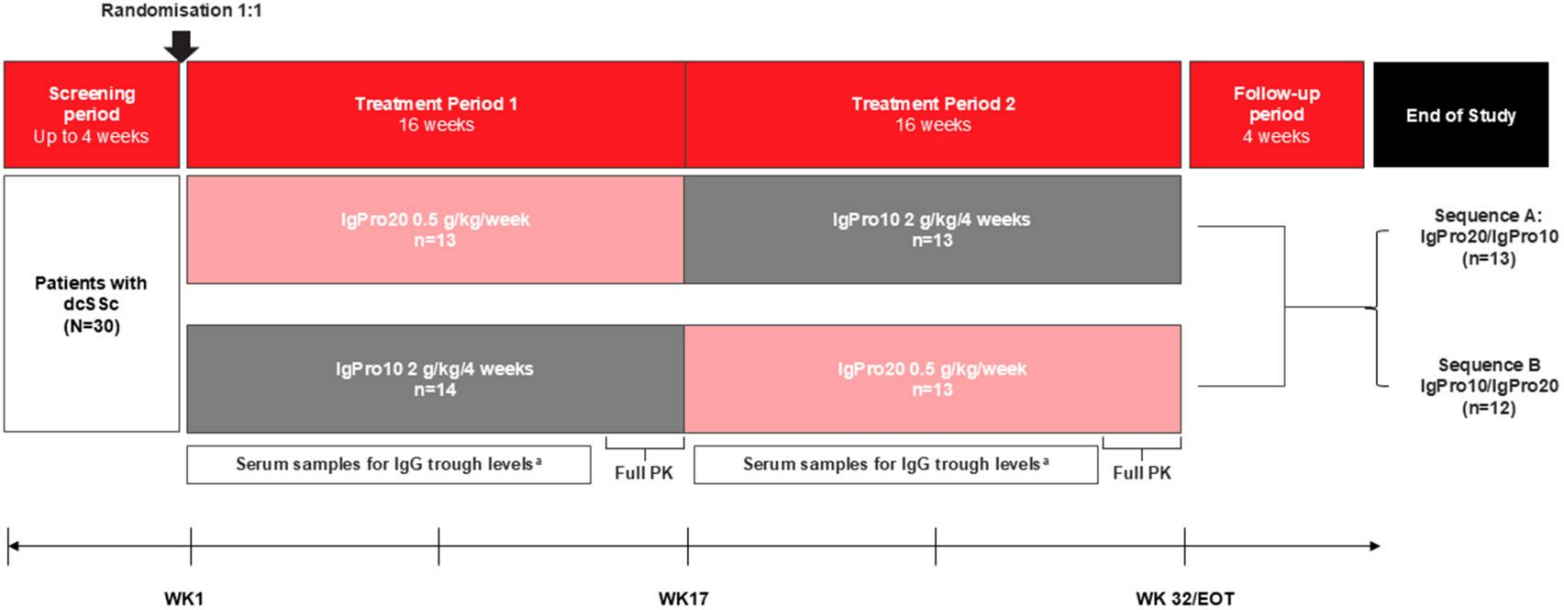
Study design. ^a^Several PK samples to measure IgG trough concentrations were collected to assess the steady state of IgPro20 or IgPro10 and carry-over effects on treatment period 2 from treatment period 1. In addition, PK samples were frequently collected over the last dose period to fully characterise the PK of IgPro20 and IgPro10. EOT: end of treatment; IgPro10: 10% human IVIG, Privigen, CSL Behring; IgPro20: 20% s.c. human immunoglobulin, Hizentra, CSL Behring; PK: pharmacokinetics

**Figure 2.**
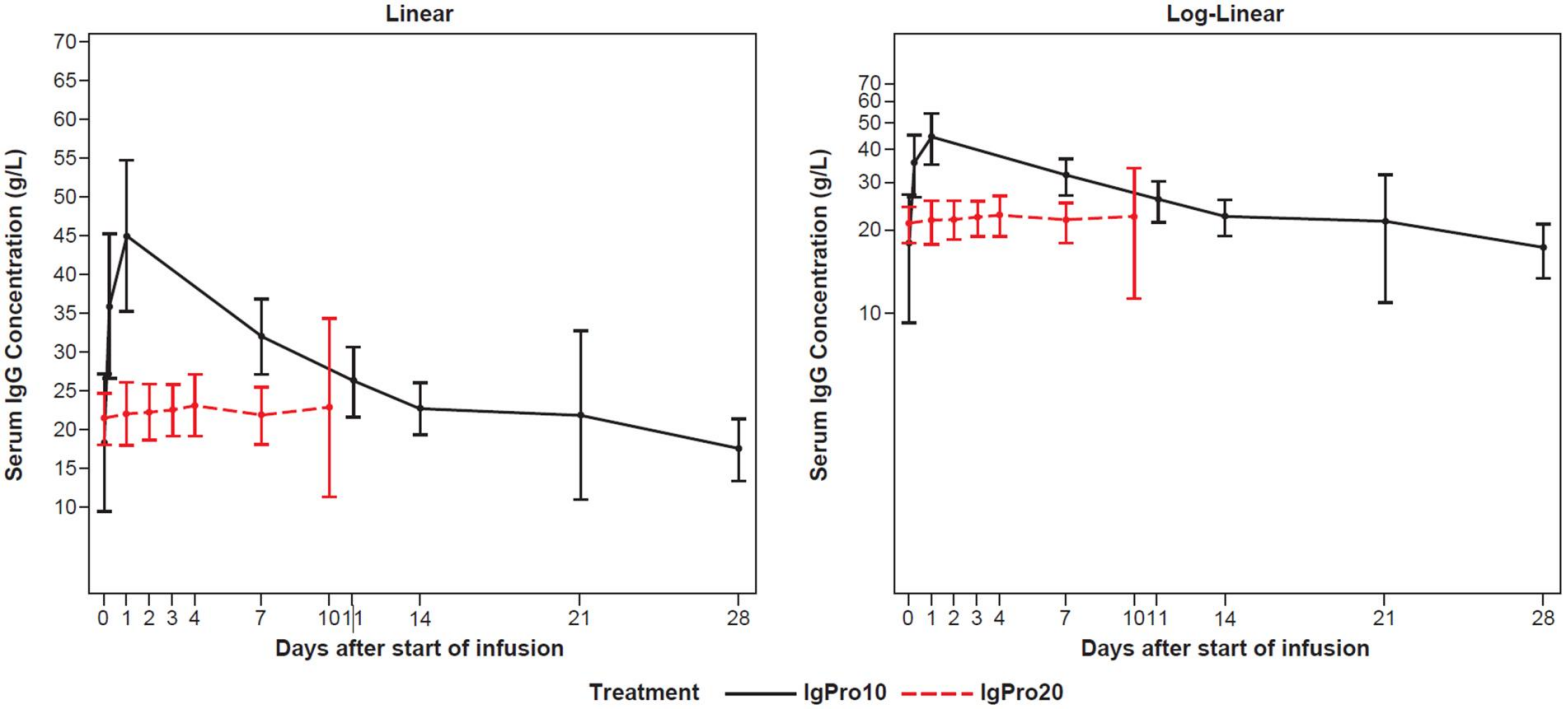
Mean (s.d.) serum IgG concentration–time profiles of patients with dcSSc following the first infusion of the last cycle of IgPro20 (0.5 g/kg/week) or IgPro10 (2 g/kg/4 weeks split over 2–5 days). IgPro10: 10% human IVIG, Privigen, CSL Behring; IgPro20: 20% s.c. human immunoglobulin, Hizentra, CSL Behring

This prospective, multicentre, randomized, open-label, crossover phase 2 study (ClinicalTrials.gov: NCT04137224; EudraCT: 2018–003149-41) was initiated at 15 study sites (Supplementary Data S1, available at *Rheumatology* online). Eligible patients were randomly assigned (1:1) to sequence A (IgPro20–IgPro10 treatment sequence) or sequence B (IgPro10–IgPro20 treatment sequence) by means of an external interactive response technology (Figs 1 and 2).

### Patient consent and involvement

Patients’ written informed consent prior to any protocol procedures was obtained and documented as per the International Council for Harmonisation of Good Clinical Practice and applicable regulatory requirements. This study was carried out in accordance with the International Council for Harmonisation of Good Clinical Practice guidelines, and the Declaration of Helsinki and all applicable national and local regulations. It was also approved by the Ethics Committee/Institutional Board—International Council for Harmonisation of Good Clinical Practice guidelines.

### Inclusion and exclusion criteria

The inclusion criteria were: males and females aged ≥18 years; a documented diagnosis of SSc (scleroderma) according to the EULAR and the ACR criteria for SSc [7] with diffuse cutaneous features [8]; skin thickness scores as measured by the modified Rodnan skin score (mRSS) of 15–45 at screening (on a scale from 0–51); a disease duration ≤5 years (from the first non-RP manifestation); capability of written informed consent; and adherence to all protocol requirements. Classification of dcSSc was confirmed using the criteria from LeRoy and colleagues [8]. Exclusion criteria included: primary rheumatic autoimmune disease other than dcSSc; mRSS >2 at the potential s.c. infusion sites; and/or a history of a skin condition, or clinical signs and symptoms of a chronic skin disease other than dcSSc, or clinical signs and symptoms of skin irritation (Supplementary Data S2, available at *Rheumatology* online).

### Treatment schedule, dosing and administration

Two treatment periods (treatment period 1 and treatment period 2; 16 weeks each) were completed by each patient, with up to 40 weeks (including screening) of study duration for an individual (Fig. 1). In sequence A, patients received a total dose of 0.5 g/kg of IgPro20 (Hizentra, CSL Behring) over two sessions per week in treatment period 1, followed by a total dose of 2 g/kg of IgPro10 (Privigen, CSL Behring) over two to five sessions on consecutive days every 4 weeks in treatment period 2 (Fig. 1). In sequence B, patients received the same treatment regimen, in the reverse order to sequence A. A follow-up visit 4 weeks after the last dose was organized for all patients who completed the study or discontinued early (Fig. 1).

The dosage of IgPro10 (2 g/kg/4 weeks) was based on the general recommendations for IVIG dosage in autoimmune conditions [13, 14] and on previous studies of IVIG in patients with SSc [21, 34]. The dosage of IgPro20 (0.5 g/kg/week) was calculated based on the 1:1 IVIG to SCIG ratio used in other autoimmune indications such as chronic inflammatory demyelinating polyneuropathy (CIDP) [35]. The total dose/volume of IgPro20 and IgPro10 were calculated based on bodyweight; however, patients weighing ≥100 kg received a fixed dose of 50 g of IgPro20 every week or 200 g of IgPro10 every 4 weeks.

The s.c. infusions were performed at selected infusion sites (e.g. on the abdomen, thighs and/or lateral hip) on skin areas with mRSS ≤2.

### Safety of IgPro20 and IgPro10

The safety of IgPro20 and IgPro10 were the primary and secondary objectives of this study, respectively. This included recording AEs (total, severity, causality and outcome) such as TEAEs, serious adverse events (SAEs), AESIs (defined as haemolysis, thromboembolic events and acute renal injury) and ISRs. The number of events, along with the number and percentage of patients with TEAEs, SAEs, AESIs and ISRs, were recorded. The median [interquartile range (IQR)] duration of ISRs, onset of ISR since the start of treatment period and time to onset of ISR since the start of last infusion were recorded. The percentage of patients with AEs were compared by treatment, treatment sequence, combination of treatment and treatment period throughout. Baseline and safety assessments were performed using the following clinical tests: physical examination, ECGs, measurement of vital signs, laboratory tests (urine, serum, haematology, virology, haemolysis), pulmonary function test(s) (PFTs) and bodyweight. Reasons for infusion interruptions were also collected. The denominator for all safety analyses was the safety analysis set (SAF), defined as ‘all patients who received at least one partial infusion of IgPro20 or IgPro10’ (*N* = 27).

### IgPro20 infusions

Data regarding the total administered volume, total dose, maximum infusion rate per infusion site, and maximum volume per infusion site was collected for IgPro20 infusions.

### PK of IgPro20 and IgPro10 

For PK assessments, serum samples were collected throughout to measure IgG trough levels and additional blood samples for rich PK sampling of IgG levels were collected at the end of each treatment period to calculate PK parameters (Fig. 1). PK parameters included: area under the concentration–time curve from time point 0 to tau (limited to the end of a dosing interval, AUC_0-tau_), baseline-corrected AUC_0-tau_, area under the concentration–time curve from time point 0 to the last quantifiable time point (AUC_0-last_) and maximum IgG concentration (C_max_).

Population bioavailability of IgPro20 relative to IgPro10 was assessed using mixed model repeated measures on a log-transformed dose-normalized baseline-corrected AUC_0-tau_. The model included treatment, treatment period and treatment-by-treatment-period interaction as fixed effects with an unstructured covariance matrix. The geometric mean ratio and corresponding 90% CI derived from the statistical model were used to assess relative bioavailability of IgPro20 compared IgPro10 based on dose-normalized baseline-corrected AUC_0-tau_.

### mRSS

An exploratory efficacy objective of this study was improvement in skin thickness following IgPro20 and IgPro10, measured by mRSS (total score and response). The mRSS was assessed at baseline, weeks 1 and 17, and end of treatment. The mRSS response definition of change was from reference visit ≤ –5 and percentage change from reference visit ≤ –25%.

### Statistical analysis

The sample size was based on feasibility, not driven by power calculations for statistical hypothesis testing. All safety analyses were based on the SAF, no formal hypothesis testing was performed. Changes from baseline were analysed by treatment, by sequence and combination of treatment and treatment period.

Population bioavailability was assessed using mixed model repeated measures on log-transformed dose-normalized baseline-corrected AUC_0-tau_. The model included treatment, treatment period and treatment-by-treatment-period interaction as fixed effects with an unstructured covariance matrix. Geometric mean ratio and corresponding 90% CI derived from the statistical model were used to assess the relative bioavailability of IgPro20 based on dose-normalized baseline-corrected AUC_0-tau_.

## Results

### Patient characteristics

The first patient was enrolled on 19 September 2019 and the last patient visit occurred on 17 May 2022. Out of 30 patients screened, 27 (90.0%) patients were randomized and treated (SAF). In total, 25 (92.6%) patients completed the study, with one patient withdrawing from the study due to moving abroad and one patient withdrawing because of a TEAE (myocardial ischaemia) (Supplementary Fig. S1, available at *Rheumatology* online). Overall, 26 patients received IgPro20, and 27 patients received IgPro10. The mean (s.d.) age of the patients was 49.3 (12.5) years; 66.7% (*N* = 18) of the patients included in the safety analysis were female and 96.3% (*N* = 26) of the patients were Caucasian (Table 1). All patients had been previously diagnosed with dcSSc and the mean (s.d.) time since diagnosis was 19.2 (16.7) months. Overall, 22 (81.5%) patients were on at least one background therapy/immunosuppressant at baseline (e.g. 15 patients were MMF at baseline). Skin involvement was moderate to severe with a mean mRSS total score (s.d.) at baseline of 24.4 (6.4) points; medical history of interstitial lung disease (ILD) was recorded for seven patients (25.9%, Table 1).

**Table 1.** Patient characteristics

Characteristic	Sequence A (IgPro20/IgPro10, *N* = 13)	Sequence B (IgPro10/IgPro20, *N* = 14)	IgPro10 (*N* = 27)	IgPro20 (*N* = 26)	Total (*N* = 27)
Age (years), mean (s.d.)	51.4 (12.2)	47.4 (12.9)	49.3 (12.5)	50.0 (12.2)	49.3 (12.5)
Sex, *n* (%)					
Female	10 (76.9)	8 (57.1)	18 (66.7)	18 (69.2)	18 (66.7)
Male	3 (23.1)	6 (42.9)	9 (33.3)	8 (30.8)	9 (33.3)
Race, *n* (%)					
Caucasian	13 (100.0)	13 (92.9)	26 (96.3)	25 (96.2)	26 (96.3)
Other	0	1 (7.1)	1 (3.7)	1 (3.8)	1 (3.7)
Weight (kg) at baseline, mean (s.d.)	78.9 (23.1)	72.6 (12.4)	75.6 (18.3)	74.8 (18.1)	75.6 (18.3)
Country, *n* (%)					
Poland	9 (69.2)	8 (57.1)	17 (63.0)	16 (61.5)	17 (63.0)
UK	3 (23.1)	3 (21.4)	6 (22.2)	6 (23.1)	6 (22.2)
Australia	0	2 (14.3)	2 (7.4)	2 (7.7)	2 (7.4)
Germany	0	1 (7.1)	1 (3.7)	1 (3.8)	1 (3.7)
Italy	1 (7.7)	0	1 (3.7)	1 (3.8)	1 (3.7)
Duration since diagnosis of SSc (months), mean (s.d.)	16.7 (13.3)	21.5 (19.6)	19.2 (16.7)	17.7 (15.2)	19.2 (16.7)
Duration since first RP (months), mean (s.d.)	70.6 (131.3)	30.0 (23.4)	50.3 (94.7)	49.9 (96.6)	50.3 (94.7)
Duration since first non-RP manifestation (months), mean (s.d.)	21.9 (13.9)	24.4 (21.2)	23.23 (17.8)	21.9 (16.8)	23.2 (17.8)
Number of background therapies/immunosuppressants at baseline, *n* (%)					
0	2 (15.4)	3 (21.4)	5 (18.5)	5 (19.2)	5 (18.5)
≥1	11 (84.6)	11 (78.6)	22 (81.5)	21 (80.8)	22 (81.5)
1	3 (23.1)	6 (42.9)	9 (33.3)	8 (30.8)	9 (33.3)
2	7 (53.8)	4 (28.6)	11 (40.7)	11 (42.3)	11 (40.7)
3	1 (7.7)	0	1 (3.7)	1 (3.8)	1 (3.7)
4	0	1 (7.1)	1 (3.7)	1 (3.8)	1 (3.7)
Any medical history event, *n* (%)	7 (53.8)	7 (50.0)	14 (51.9)	14 (53.8)	14 (51.9)
Any prior medication, *n* (%)	9 (69.2)	10 (71.4)	19 (70.4)	18 (69.2)	19 (70.4)
Any concomitant medication, *n* (%)	13 (100.0)	14 (100.0)	27 (100.0)	26 (100.0)	27 (100.0)
Baseline mRSS total score, mean (s.d.)	23.8 (5.8)	25.0 (7.1)			24.4 (6.4)
Any concomitant disease, *n* (%)	12 (92.3)	13 (92.9)	25 (92.6)	24 (92.3)	25 (92.6)
Vascular disorders	8 (61.5)	5 (35.7)	13 (48.1)	12 (46.2)	13 (48.1)
Musculoskeletal and connective tissue disorders	5 (38.5)	6 (42.9)	11 (40.7)	11 (42.3)	11 (40.7)
Gastrointestinal disorders	6 (46.2)	4 (28.6)	10 (37.0)	10 (38.5)	10 (37.0)
Respiratory, thoracic and mediastinal disorders	6 (46.2)	4 (28.6)	10 (37.0)	9 (34.6)	10 (37.0)
Interstitial lung disease	4 (30.8)	3 (21.4)	7 (25.9)	6 (23.1)	7 (25.9)
Endocrine disorders	5 (38.5)	4 (28.6)	9 (33.3)	9 (34.6)	9 (33.3)
Metabolism and nutrition disorders	4 (30.8)	4 (28.6)	8 (29.6)	8 (30.8)	8 (29.6)
Reproductive system and breast disorders	5 (38.5)	3 (21.4)	8 (29.6)	8 (30.8)	8 (29.6)

In sequence A, patients received a total dose of 0.5 g/kg of IgPro20 over two sessions per week in treatment period 1, and a total dose of 2 g/kg of IgPro10 over two to five sessions on consecutive days every 4 weeks in treatment period 2. In sequence B, patients received the same treatment regimen, but vice versa. mRSS: modified Rodnan skin score; IgPro10: 10% human IVIG, Privigen, CSL Behring; IgPro20: 20% s.c. human immunoglobulin, Hizentra, CSL Behring; *N*: number of patients.

### Safety of IgPro20 and IgPro10

#### TEAEs

In total, 22 patients (81.5%) experienced 107 TEAEs, the majority of which were mild or moderate (102 events, Table 2); with 18 patients (69.2%) experiencing 49 TEAEs on IgPro20 and 13 patients (48.1%) experiencing 58 TEAEs on IgPro10. The most common TEAEs (>10% of all patients) by preferred term were headaches [IgPro20 treatment periods: one patient (3.8%) with one event; IgPro10 treatment periods: five patients (18.5%) and 11 events], coronavirus disease 2019 (COVID-19) [three patients (11.5%) and three events in IgPro20 treatment periods only], diarrhoea [IgPro20 treatment periods: one patient (3.8%) with one event; IgPro10 treatment periods: two patients (7.4%)] and two events) and vomiting [IgPro20 treatment periods: one patient (3.8%) with one event; IgPro10 treatment periods: two patients (7.4%) and two events].

**Table 2.** TEAEs and treatment-related TEAEs recorded

	Period 1				Period 2				IgPro10 periods (*N* = 27)		IgPro20 periods (*N* = 26)		Total (*N* = 27)	
	IgPro10 (*N* = 14)		IgPro20 (*N* = 13)		IgPro10 (*N* = 13)		IgPro20 (*N* = 13)							
	*n* (%)	E	*n* (%)	E	*n* (%)	E	*n* (%)	E	*n* (%)	E	*n* (%)	E	*n* (%)	E
TEAE by severity														
Mild	8 (57.1)	40	4 (30.8)	11	5 (38.5)	10	6 (46.2)	21	13 (48.1)	50	10 (38.5)	32	14 (51.9)	82
Moderate	4 (28.6)	4	7 (53.8)	7	2 (15.4)	3	3 (23.1)	6	6 (22.2)	7	10 (38.5)	13	13 (48.1)	20
Severe	1 (7.1)	1	0	0	0	0	3 (23.1)	4	1 (3.7)	1	3 (11.5)	4	4 (14.8)	5
TEAEs related to study treatment														
Any TEAE	8 (57.1)	24	4 (30.8)	8	3 (23.1)	3	4 (30.8)	17	11 (40.7)	27	8 (30.8)	25	15 (55.6)	52
Serious TEAE	0	0	0	0	0	0	0	0	0	0	0	0	0	0
TEAE resulting in death	0	0	0	0	0	0	0	0	0	0	0	0	0	0
TEAE leading to discontinuation of study treatment	0	0	0	0	0	0	0	0	0	0	0	0	0	0
TEAE leading to interruption or withdrawal														
TEAE leading to dose interruptions	2 (14.3)	2	4 (30.8)	6	0	0	2 (15.4)	2	2 (7.4)	2	6 (23.1)	8	8 (29.6)	10
TEAE leading to study withdrawal	0	0	0	0	0	0	1 (7.7)	1	0	0	1 (3.8)	1	1 (3.7)	1
Detailed treatment-related TEAES														
ISRs	0	0	2 (15.4)	2	0	0	3 (23.1)	12	0	0	5 (19.2)	14	5 (18.5)	14
Nervous system disorders	6 (42.9)	11	1 (7.7)	2	0	0	1 (7.7)	2	6 (22.2)	11	2 (7.7)	4	7 (25.9)	15
General disorders and administration site conditions	1 (7.1)	1	2 (15.4)	2	0	0	3 (23.1)	12	1 (3.7)	1	5 (19.2)	14	6 (22.2)	15
Skin and subcutaneous tissue disorders	2 (14.3)	3	1 (7.7)	1	3 (23.1)	3	0	0	5 (18.5)	6	1 (3.8)	1	6 (22.2)	7
Gastrointestinal disorders	3 (21.4)	5	2 (15.4)	3	0	0	1 (7.7)	1	3 (11.1)	5	3 (11.5)	4	5 (18.5)	9
Respiratory, thoracic and mediastinal disorders	1 (7.1)	2	0	0	0	0	1 (7.7)	1	1 (3.7)	2	1 (3.8)	1	2 (7.4)	3
Hepatobiliary disorders	1 (7.1)	1	0	0	0	0	0	0	1 (3.7)	1	0	0	1 (3.7)	1
Injury, poisoning and procedural complications	0	0	0	0	0	0	1 (7.7)	1	0	0	1 (3.8)	1	1 (3.7)	1
Renal and urinary disorders	1 (7.1)	1	0	0	0	0	0	0	1 (3.7)	1	0	0	1 (3.7)	1

In period 1, patients were assigned to 16 weeks of IgPro20 (0.5 g/kg/week) or IgPro10 (2 g/kg/4 weeks split over 2–5 days). Patients then received the alternative treatment during period 2. E: number of events; IgPro10: 10% human IVIG, Privigen, CSL Behring; IgPro20: 20% s.c. human immunoglobulin, Hizentra, CSL Behring; ISRs: infusion site reactions; *N*/*n*: number of patients; TEAE: treatment-emergent adverse events.

In total, 15 patients (55.6%) experienced 52 TEAEs considered related to the study treatment (Table 2). The most common (>10% of all patients) treatment-related TEAE by preferred term was headache [one patient (3.8%) with one event on IgPro20; five patients (18.5%) and 10 events on IgPro10, Table 2]. Overall, four patients (14.8%) had severe TEAEs (five events); in the IgPro20 treatment periods, three patients (11.5%) experienced four of the total five events and one patient (3.7%) experienced one severe TEAE in an IgPro10 treatment period (Table 2).

#### SAEs

A total of six patients (22.2%) experienced 10 SAEs, none of which was considered related to study treatment (Supplementary Table S1, available at *Rheumatology* online). During the IgPro20 treatment periods, five patients (19.2%) experienced six SAEs (upper gastrointestinal haemorrhage, chest pain, myocardial infarction, myocardial ischaemia, breast cancer and ILD). During the IgPro10 treatment periods, two patients (7.4%) experienced four SAEs (viral infection, chronic gastritis, vomiting and dehydration). One patient (3.8%) had two SAEs on IgPro20 (myocardial ischaemia and myocardial infarction) and was discontinued from the study (Supplementary Table S1, available at *Rheumatology* online).

#### AESIs and TEAEs leading to treatment discontinuation

One grade 1 myocardial infarction was reported during the study as an AESI; this occurred during IgPro20 treatment and was judged unrelated to treatment by the investigator. One TEAE of COVID-19 led a patient to discontinue treatment in period 1; once recovered, the patient went on to complete the study. No other AESIs or TEAEs leading to discontinuation of the study were reported. No deaths were reported in the study.

#### Reasons for interruptions

In total, there were 10 TEAEs (occurring in eight patients, 29.6%) that led to study drug interruptions. Two patients (7.4%) had two TEAEs leading to study drug interruptions in the IgPro10 periods (upper respiratory tract infection and viral infection; both in period 1) and six patients (23.1%) had eight events in the IgPro20 periods (rash, upper gastrointestinal haemorrhage, abdominal distension, abdominal pain, lower respiratory tract infection, breast cancer and two COVID-19 events; six in period 1 and two in period 2). All of the TEAEs that led to drug interruptions were unrelated to the study treatment except for rash, abdominal distension, and abdominal pain. No ISRs led to study drug interruptions.

In addition to TEAEs, infusions were interrupted due to user error with the pump during the IgPro20 infusion (two events in two patients), pump malfunction during IgPro20 infusions (two events in two patients), to comply with maximum IgPro20 infusion rate and volume in the protocol (one event in one patient), IgPro10 vial/bottle changes (eight events in one patient), and the patient needing the bathroom during an IgPro10 infusion (one event in one patient). Additional difficulties during administration which did not result in an interruption occurred for one patient during the IgPro20 infusions, these included further pump malfunctions, incomplete supplies, and a syringe malfunction.

#### ISRs

Overall, five patients (18.5%) experienced 14 ISRs following IgPro20 infusions, with seven ISR events reported in one patient and one ISR event reported as not recovered/not resolved (Table 3). All events were related to the study treatment and were mild/moderate in severity. No ISRs led to discontinuation of the study treatment or study withdrawal. Infusion-site pain and infusion-site swelling were the most common ISRs [two patients (7.4%) experienced three events each, Table 2 and Supplementary Table S2, available at *Rheumatology* online]. Overall, 686 IgPro20 infusions were performed, resulting in an overall ISR rate per infusion of 0.02, i.e. two ISRs per 100 infusions. The median (IQR) time to onset of ISR since the start of last infusion was 78.0 (30.0–105.0) min. The median (IQR) duration of ISR was 220.0 (162.0–1170.0) min. No ISRs were reported in patients receiving IgPro10 infusions.

**Table 3.** ISRs: in period 1, patients were assigned to 16 weeks of IgPro20 (0.5 g/kg/week) or IgPro10 (2 g/kg/4 weeks split over 2–5 days)

	Period 1				Period 2				IgPro10 periods (*N* = 27)		IgPro20 periods (*N* = 26)		Total (*N* = 27)	
	IgPro10 (*N* = 14)		IgPro20 (*N* = 13)		IgPro10 (*N* = 13)		IgPro20 (*N* = 13)							
	*n* (%)	E	*n* (%)	E	*n* (%)	E	*n* (%)	E	*n* (%)	E	*n* (%)	E	*n* (%)	E
Any ISR	0	0	2 (15.4)	2	0	0	3 (23.1)	12	0	0	5 (19.2)	14	5 (18.5)	14
ISR leading to dose interruptions	0	0	0	0	0	0	0	0	0	0	0	0	0	0
ISR leading to discontinuation of study treatment	0	0	0	0	0	0	0	0	0	0	0	0	0	0
ISR leading to study withdrawal	0	0	0	0	0	0	0	0	0	0	0	0	0	0
ISR related to study treatment	0	0	2 (15.4)	2	0	0	3 (23.1)	12	0	0	5 (19.2)	14	5 (18.5)	14
ISR by severity														
Mild	0	0	1 (7.7)	1	0	0	3 (23.1)	6	0	0	4 (15.4)	7	4 (14.8)	7
Moderate	0	0	1 (7.7)	1	0	0	3 (23.1)	6	0	0	4 (15.4)	7	4 (14.8)	7
Severe	0	0	0	0	0	0	0	0	0	0	0	0	0	0
Outcome of ISR														
Recovered/resolved	0	0	2 (15.4)	2	0	0	3 (23.1)	11	0	0	5 (19.2)	13	5 (18.5)	13
Not recovered/not resolved	0	0	0	0	0	0	1 (7.7)	1	0	0	1 (3.8)	1	1 (3.7)	1

Patients then received the alternative treatment during period 2.

E: number of events; IgPro10: 10% human IVIG, Privigen, CSL Behring; IgPro20: 20% s.c. human immunoglobulin, Hizentra, CSL Behring; ISR: infusion site reaction; *N*/*n*: number of patients.

#### Clinical tests

During the course of the study, no clinically relevant changes in mean data were observed for physical examination, vital signs, body weight, clinical laboratory tests, ECGs or PFTs (data not shown for brevity).

#### IgPro20 infusions

Per patient (*n* = 26), the mean (s.d.) total administered volume of IgPro20 was 2431.4 (731.5) ml and the total dose (g) was 486.3 (146.3) g. The mean (s.d.) maximum volume per infusion site was 43.1 (13.6) ml/site and the maximum infusion rate per infusion site was 42.5 (13.9) ml/h/site.

### PK of IgPro20 and IgPro10

At week 1 (baseline), the mean (s.d.) trough serum IgG concentrations were 13.3 (4.3) g/l and 12.3 (4.3) g/l in sequence A (IgPro20/IgPro10) and sequence B (IgPro10/IgPro20), respectively. Overall mean (s.d.) trough serum IgG concentrations following administration of IgPro20 ranged from 22.2 (4.1) to 23.8 (12.9) g/l in treatment period 1, and from 20.6 (2.0) to 22.0 (2.7) g/l in treatment period 2, which was numerically higher than following IgPro10 treatment at 17.3 (2.9)–17.9 (2.8) g/l in treatment period 1 and 17.1 (6.7)–19.6 (11.6) g/l in treatment period 2. As expected, the mean (s.d.) C_max_ was numerically lower following IgPro20 administration [23.7 (1.2) g/l] compared with IgPro10 administration [46.1 (1.2) g/l] (Fig. 2). Further PK parameters (AUC_0-tau_, baseline corrected _AUC0-tau_, AUC_0-last_ and C_max_) are presented in Table 4. The geometric mean (geometric s.d.) dose-normalized, baseline-corrected AUC_0-tau_ were 44.8 (1.4) h*g/l for IgPro20 and 60.2 (1.4) h*g/l for IgPro10 (Table 4).

**Table 4.** Pharmacokinetic parameters of IgPro20 and IgPro10, and relative bioavailability of IgPro20

	IgPro10		IgPro20		Relative bioavailability
	*n*	Geometric mean (geometric s.d.)	*n*	Geometric mean (geometric s.d.)	Geometric mean ratio (90% CI)
AUC_0-tau_ (h*g/L)					
Period 1	12	17672.0 (1.1)	12	3835.7 (1.2)	
Period 2	12	16942.7 (1.2)	11	3581.1 (1.1)	
Overall	24	17303.5 (1.2)	23	3711.7 (1.2)	
Baseline-corrected AUC_0-tau_ (h*g/L)					
Period 1	12	9895.0 (1.3)	12	1539.4 (1.3)	
Period 2	12	8021.4 (1.3)	12	1648.7 (1.4)	
Overall	24	8909.0 (1.3)	24	1593.1 (1.4)	
AUC_0-last_ (h*g/L)					
Period 1	12	17349.7 (1.1)	12	5361.6 (1.2)	
Period 2	13	16520.5 (1.2)	12	4898.0 (1.2)	
Overall	25	16913.5 (1.2)	24	5124.6 (1.2)	
C_max_ (g/L)					
Period 1	12	45.0 (1.2)	12	24.2 (1.2)	
Period 2	13	47.1 (1.2)	12	23.2 (1.1)	
Overall	25	46.1 (1.2)	24	23.7 (1.2)	
Dose-normalized baseline-corrected AUC_0-tau_ (h*g/L)					
Sequence A^a^	12	52.0 (1.4)	12	42.0 (1.5)	0.831 (0.734, 0.940)
Sequence B^b^	12	69.7 (1.3)	12	47.8 (1.3)	0.698 (0.624, 0.780)
Overall	24	60.2 (1.4)	24	44.8 (1.4)	0.761 (0.703, 0.823)

a IgPro20 followed by IgPro10 treatment sequence;  ^b^  IgPro10 followed by IgPro20 treatment sequence. AUC_0-tau_: area under the concentration–time curve from time point 0 to tau (limited to the end of a dosing interval); AUC_0-last_: baseline-corrected AUC_0-tau_, area under the concentration–time curve from time point 0 to the last quantifiable time point; C_max_: maximum IgG concentration; IgPro10: 10% human IVIG, Privigen, CSL Behring; IgPro20: 20% s.c. human immunoglobulin, Hizentra, CSL Behring.

### Bioavailability

The bioavailability (90% CI) of IgPro20 relative to IgPro10 was numerically higher in sequence A [0.831 (0.734, 0.940)] than in sequence B [0.698 (0.624, 0.780)]; the overall population relative bioavailability of IgPro20 was 0.761 (0.703, 0.823), i.e. 76.1% bioavailability relative to IgPro10 (Table 4).

### mRSS

Improvements in mean mRSS total score were observed following each treatment period as measured at week 17 and week 32 (Supplementary Table S3, available at *Rheumatology* online). In total, 11 (40.7%) patients were mRSS responders in Weeks 1–17, and 18 (66.7%) were responders over Weeks 1–32 (Supplementary Table S3, available at *Rheumatology* online).

## Discussion

This multicentre, randomized, open-label, crossover, phase 2 study evaluated for the first time, the safety, PK and bioavailability of IgPro20 (SCIG, Hizentra, CSL Behring) in adults with dcSSc. S.c. administration of IgPro20 in patients with dcSSc associated with moderate-to-severe skin thickness is well tolerated with acceptable safety, PK and bioavailability profiles. The results also indicate acceptable safety and PK profiles for IgPro10 (IVIG, Privigen, CSL Behring) in adults with dcSSc.

The safety profile of IgPro20 observed here is consistent with the established safety profiles for other approved indications such as CIDP [17–20]. Overall, the majority of TEAEs were mild or moderate in severity, and approximately half were considered related to study treatment by the investigator. None of the SAEs was considered related to study treatment. Overall, the ISR rate was low and considered comparable to the incidence of IgPro20 ISRs in other immuno-modulatory indications [20]. No clinically relevant concerning trends were observed for vital signs, bodyweight, clinical laboratory tests, ECGs or PFTs. This study indicated that s.c. IgPro20 infusions at a total dose of 0.5 g/kg every week (considered as a high immunomodulatory dose) was well tolerated in this patient group presenting with pathologically changed skin and s.c. tissues.

This work was the first study to explore PK of IgG in patients with dcSSc. As expected, when comparing administration methods (SCIG and IVIG) and their associated dosing regimens, C_max_ and geometric mean dose-normalized baseline-corrected AUC_0-tau_ were higher following i.v. administration, as IVIG initially provides a large peak followed by a ‘wear-off’ of immunoglobulin levels, whereas SCIG provides lower but more stable levels [28, 36]. Overall, the IgPro20 PK profiles observed in patients with dcSSc were similar to those observed in other indications with no skin-thickening symptoms (such as primary immunodeficiency and CIDP) [17, 18, 20]. This finding indicates that the disease state did not affect overall PK or bioavailability of human IgG given by the s.c. route. Moreover, the bioavailability of IgPro20 was comparable to the bioavailability of IgPro20 in other indications (53–79%) [17, 19, 20] and to the reported bioavailability of other SCIG products on the market (65–69%) [37].

The secondary objectives were to evaluate the use of IVIG in patients with dcSSc and the clinical data demonstrated that the observed safety and PK profiles are similar to those observed for other approved IgPro10 indications [19].

The pathological features of dcSSc raise the question of the feasibility of s.c. administration of IgG in these patients. The small vessel vasculopathy, lymphatic vessel attenuation and excessive collagen deposition in the skin and internal organs [2, 32, 33] might interfere with absorption of SCIG through the lymph vessels into the bloodstream or increase the risks for AEs. The results obtained here constitute a proof-of-concept that safety, PK and bioavailability profiles, following administration of SCIG in patients with SSc, are comparable to those observed in other indications with no skin pathology [17–20, 38].

For patients with dcSSc, SCIG might allow an easier administration than IVIG as SCIG can be self-administered at home with a shorter infusion time and more flexible dosing regimens [29]. With dcSSc disease strongly affecting the patient’s quality of life [10, 39], an easier administration, and reduced need to have IgG administered in infusion centres, might be particularly advantageous [40]. Furthermore, safety profiles established in approved indications show that compared with IVIGs, SCIGs are characterized by a lower rate of systemic AEs making SCIG a more attractive therapeutic option for patients [27, 41]. Finally, SCIG could be an option for patients in whom IVIG infusions are difficult due to i.v. access issues.

The initial efficacy endpoint (mRSS) explored during the study reveals potential improvement following treatment for both sequence A and B. However, further efficacy analyses are required.

The limitations for evaluation of efficacy in this study include the lack of a washout period in the crossover design and lack of a control arm. Furthermore, as the patient population was mostly Caucasian, broader data are required to ensure these results can be generalized. The study only included s.c. infusions performed on skin areas with mRSS ≤2, therefore the results are unable to confirm whether s.c. infusions are impacted by increased skin thickness associated with SSc. Exploratory endpoints included ACR Composite Response Index in dcSSc, mRSS, physician global assessment, HAQ disability index including scleroderma, patient global assessment and forced vital capacity predicted. Results from these exploratory efficacy endpoints are to be published in a subsequent publication. A few previous open-label and observational studies have explored the effects of IVIG on the symptoms of patients with SSc and reported improvement [16, 21–23, 25, 42].

In conclusion, this study showed that s.c. administration of immunoglobulin is generally well tolerated in patients with dcSSc. The ISR rate was low with no severe or serious TEAEs affecting the skin reported, despite moderate-to-severe skin involvement in all subjects and pathological skin features. Overall safety, PK and bioavailability profiles of IgPro20, and safety and PK of IgPro10 were similar to those observed in other indications.

## Supplementary Material

keaf066_Supplementary_Data

## Data Availability

Data are available upon reasonable request. CSL Behring’s policy on data sharing can be found at https://www.csl.com/research-and-development/clinical-studies/research-practices.
