# Supplementary material for: A phase 2 randomized trial of safety and pharmacokinetics of IgPro20 and IgPro10 in patients with diffuse cutaneous systemic sclerosis
Source: Rheumatology (Oxford). 2025 Feb 5;64(6):3657–66. doi: 10.1093/rheumatology/keaf066 (PMC12107079; doi:10.1093/rheumatology/keaf066)
Supplement: keaf066_Supplementary_Data [file keaf066_supplementary_data.docx]

**Supplemental Materials**

**Supplementary Data S1.** Patient recruitment centres

| **Site** |
| --- |
| Royal Adelaide Hospital  Port Rd.  Adelaide  South Australia  5000  Australia |
| Vincent's Hospital Fitzroy  (Melbourne)  41 Victoria Parade  Fitzroy  Melbourne  3065  Australia |
| Hôpital Cochin  27 Rue Du Faubourg Saint-Jacques  14E Arrondissement  Paris  75014  France |
| Uniklinik Köln, Studienzentrum der Klinik  1 für lnnere Medizin  Kerpener Strae 62  Köln  50937  Germany |
| Klinikverbund St. Antonius u. St. Josef  Bergstraße 6-12  Wuppertal  42105  Germany |
| Charité – Universitätsmedizin Berlin  Chariteplatz 1  Berlin  10117  Germany |
| University of L'Aquila  Via dell'Ospedale  Delta 6 Bldg.  L'Aquila  67100  Italy |
| ASST degli Spedali Civili di Brescia  Piazzale Spedali Civili 1  Brescia  25123  Italy |
| Azienda Ospedaliera Gaetano Pini  Piazza Cardinal Ferrari 1  Milano  20122  Italy |
| AOU Careggi, Rheumatology Unit  Via delle Oblate 4  Firenze  50134  Italy |
| Università Politecnica delle Marche  Piazza Roma 22  Ancona  60121  Italy |
| Szpital Kliniczny Dzieciatka Jezus  Koszykowa 82 A  Warsaw  02-008  Poland |
| Narodowy Instytut Geriatrii,  Reumatologii i Rehabilitacji  Spartańska 1  Warsaw  02-637  Poland |
| Uniwersytecki Szpital Kliniczny W  Bialymstoku  ul. M Sklodowskiej-Curie 24A  Bialystok  15-276  Poland |
| The Royal Free London NHS  Foundation Trust - The Royal Free  Hospital  Pond Street  London  NW3 2QG  UK |

**Supplementary Data S2.** Complete list of the study exclusion criteria

Patients were not enrolled into the study if they met any of the following exclusion criteria:

1. Primary rheumatic autoimmune disease other than dcSSc, including but not limited to rheumatoid arthritis, systemic lupus erythematosus, mixed connective tissue disorder, polymyositis, or dermatomyositis, as determined by the investigator. Note: patients with fibromyalgia, secondary Sjogren’s syndrome, and scleroderma-associated myopathy at screening were not excluded
2. Patient had mRSS >2 at the potential SC infusion sites
3. History of skin condition or clinical signs and symptoms of a chronic skin disease other than SSc or skin manifestation of an allergic disease or other dermatological conditions precluding SC infusion at potential SC infusion sites (e.g., dermatitis, eczema, psoriasis)
4. Patient had clinical signs and symptoms of skin irritation (e.g., pruritus, burning, erythema) or hypo/hyperpigmentation (e.g., scars, tattoos) at the potential SC infusion sites
5. Significant pulmonary arterial hypertension as documented by mean pulmonary arterial pressure >30 mmHg on right heart catheterisation requiring SC or IV prostacyclin or use of dual oral therapies
6. FVC <50% predicted or a diffusing capacity of the lung for carbon dioxide (DLCO) ≤40% predicted (corrected for haemoglobin [HGB])
7. SSc renal crisis within 2 years before screening
8. Evidence of chronic kidney disease with an estimated glomerular filtration rate of
   <45 mL/min/1.73 m^2^ (as calculated by the Chronic Kidney Disease Epidemiology Collaboration equation) or if patient is receiving dialysis. Patients with current confirmed diagnosis of diabetes mellitus requiring medication with an estimated glomerular filtration rate <90 mL/min/1.73 m^2^
9. History of documented thrombotic episode e.g., pulmonary embolism, deep vein thrombosis, myocardial infarction, or thromboembolic stroke at any time (note: history of superficial thrombophlebitis is not exclusionary)
10. Known documented thrombophilia abnormalities including current blood hyper viscosity (within 4 weeks before screening), protein S or protein C deficiency, anti-thrombin-3 deficiency, plasminogen deficiency, antiphospholipid syndrome, Factor V Leiden mutation, dysfibrinogenemia, or prothrombin G20210A mutation
11. Recent surgery requiring general anaesthesia within the last 4 weeks before screening
12. Greater than three specified current risk factors for TEEs (documented and currently ongoing conditions): atrial fibrillation, coronary disease, diabetes mellitus, dyslipidaemia, hypertension, obesity (body mass index ≥ 30 kg/m2), recent significant trauma, or immobility (wheelchair-bound or bedridden)
13. Cardiac insufficiency (New York Heart Association Class III or IV), cardiomyopathy, significant persistent arrhythmia, unstable or advanced ischemic heart disease, or uncontrolled hypertension
14. Ongoing active serious infection (including, but not limited to, pneumonia, bacteraemia/septicaemia, osteomyelitis/septic arthritis, bacterial meningitis, visceral abscess) at screening or hospitalisation and/or treatment with IV antibiotics for a serious infection within 2 months before screening
15. A positive result at screening of any of the following viral markers: human immunodeficiency virus‑1/‑2, hepatitis C virus, or hepatitis B virus
16. Malignancy in the past 2 years, except for non-melanoma skin cancer, cervical carcinoma in situ, or other in situ cancer if it has been excised and treated within the past year
17. Known medical conditions whose symptoms and effects could alter protein catabolism and/or IgG utilisation (e.g., protein-losing enteropathies, nephrotic syndrome) and proteinuria (defined as albumin-to-creatinine ratio > 30 mg/g)
18. Note: Transient and clinically insignificant proteinuria as based on the investigator’s judgment should be discussed with the Medical Monitor and is not exclusionary
19. Known hyperprolinaemia type I or II
20. Known Immunoglobulin A (IgA) deficiency or serum IgA level < 5% lower limit of normal
21. History of clinically significant or uncontrolled illness that, in the opinion of the investigator, would prevent participation in the study
22. Psychiatric, addictive, or other disorders that compromise the ability to give informed consent for participating in this study. This includes patients with a recent history of abusing alcohol or illicit drugs
23. Clinically significant abnormal 12-lead ECG that, in the opinion of the investigator, would prevent participation in the study
24. Clinically significant abnormal laboratory testing at screening that, in the opinion of the investigator, would prevent participation in the study
25. Currently receiving or having received therapy not permitted during the study or during predefined windows before screening
26. Known allergic or other severe reactions to immunoglobulins or other blood products, including a history of haemolysis after IVIG infusion
27. Known or suspected antibodies to the IP or to excipients of the IP
28. A female who is pregnant, breastfeeding, or is a woman of childbearing potential who does not agree to use acceptable methods of contraception; a male who does not agree to use acceptable methods of contraception
29. Participated in another study with an investigational agent within 3 months
30. Involved in the planning and/or conduct of the study (applies to CSLB staff and dependents, staff at the study site, site examiner, or third-party vendors)
31. Individuals who have been institutionalised as a result of an official or court order
32. Any issues or conditions that would render the patient unsuitable for participation in the study.


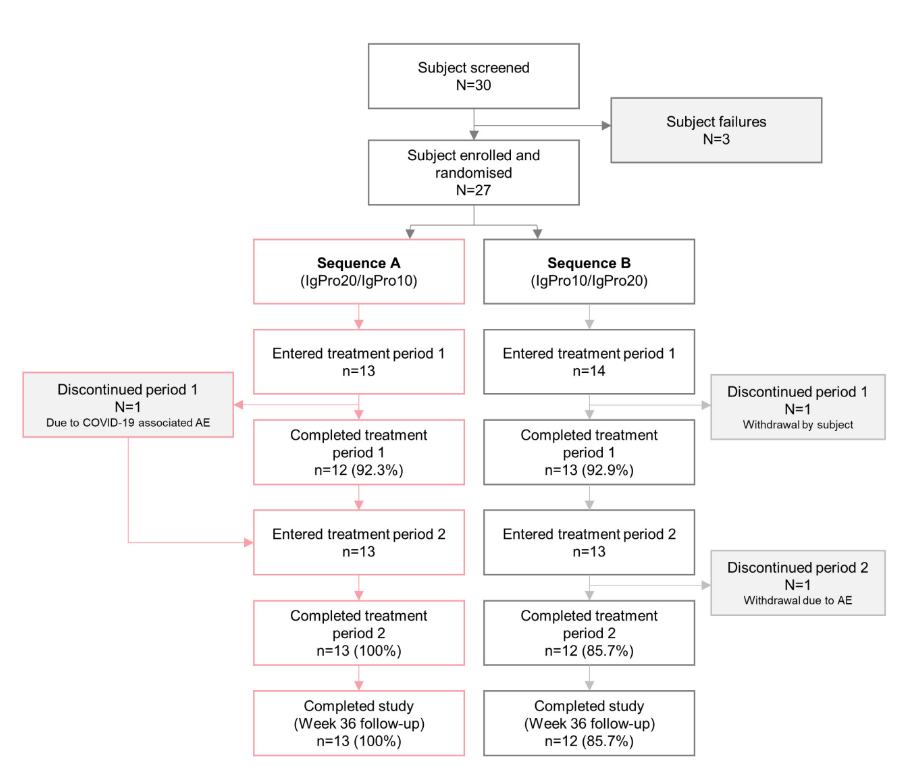


**Supplementary Figure S1.** Flowchart of patients with dcSSc throughout the study.

AE, adverse event; COVID-19, coronavirus disease 2019; dcSSc, diffuse cutaneous systemic sclerosis; IgPro10, 10% intravenous human immunoglobulin, Privigen, CSL Behring; IgPro20, 20% subcutaneous human immunoglobulin, Hizentra, CSL Behring.

**Supplementary Table S1.** Serious TEAEs by system organ class and preferred term (SAF).

|  | **Period 1** | | | | **Period 2** | | | | **IgPro10**  **Periods**  **(N=27)** | | **IgPro20**  **Periods**  **(N=26)** | |  |  |
| --- | --- | --- | --- | --- | --- | --- | --- | --- | --- | --- | --- | --- | --- | --- |
|  | **IgPro10**  **(N=14)** | | **IgPro20**  **(N=13)** | | **IgPro10**  **(N=13)** | | **IgPro20**  **(N=13)** | |  |  |  |  | **Total**  **(N=27)** | |
|  | **n (%)** | **E** | **n (%)** | **E** | **n (%)** | **E** | **n (%)** | **E** | **n (%)** | **E** | **n (%)** | **E** | **n (%)** | **E** |
| **Any serious TEAE** | 1 (7.1) | 1 | 2 (15.4) | 2 | 1 (7.7) | 3 | 3 (23.1) | 4 | 2 (7.4) | 4 | 5 (19.2) | 6 | 6 (22.2) | 10 |
| **Infusion site reactions** | 0 | 0 | 0 | 0 | 0 | 0 | 0 | 0 | 0 | 0 | 0 | 0 | 0 | 0 |
| **Thromboembolic events** | 0 | 0 | 0 | 0 | 0 | 0 | 1 (7.7) | 1 | 0 | 0 | 1 (3.8) | 1 | 1 (3.7) | 1 |
| **Myocardial infarction** | 0 | 0 | 0 | 0 | 0 | 0 | 1 (7.7) | 1 | 0 | 0 | 1 (3.8) | 1 | 1 (3.7) | 1 |
| **Cardiac disorders** | 0 | 0 | 0 | 0 | 0 | 0 | 1 (7.7) | 2 | 0 | 0 | 1 (3.8) | 2 | 1 (3.7) | 2 |
| **Myocardial infarction** | 0 | 0 | 0 | 0 | 0 | 0 | 1 (7.7) | 1 | 0 | 0 | 1 (3.8) | 1 | 1 (3.7) | 1 |
| **Myocardial ischaemia** | 0 | 0 | 0 | 0 | 0 | 0 | 1 (7.7) | 1 | 0 | 0 | 1 (3.8) | 1 | 1 (3.7) | 1 |
| **Gastrointestinal disorders** | 0 | 0 | 1 (7.7) | 1 | 1 (7.7) | 2 | 0 | 0 | 1 (3.7) | 2 | 1 (3.8) | 1 | 1 (3.7) | 3 |
| **Chronic gastritis** | 0 | 0 | 0 | 0 | 1 (7.7) | 1 | 0 | 0 | 1 (3.7) | 1 | 0 | 0 | 1 (3.7) | 1 |
| **Upper gastrointestinal haemorrhage** | 0 | 0 | 1 (7.7) | 1 | 0 | 0 | 0 | 0 | 0 | 0 | 1 (3.8) | 1 | 1 (3.7) | 1 |
| **Vomiting** | 0 | 0 | 0 | 0 | 1 (7.7) | 1 | 0 | 0 | 1 (3.7) | 1 | 0 | 0 | 1 (3.7) | 1 |
| **General disorders and administration site conditions** | 0 | 0 | 1 (7.7) | 1 | 0 | 0 | 0 | 0 | 0 | 0 | 1 (3.8) | 1 | 1 (3.7) | 1 |
| **Chest pain** | 0 | 0 | 1 (7.7) | 1 | 0 | 0 | 0 | 0 | 0 | 0 | 1 (3.8) | 1 | 1 (3.7) | 1 |
| **Infections and infestations** | 1 (7.1) | 1 | 0 | 0 | 0 | 0 | 0 | 0 | 1 (3.7) | 1 | 0 | 0 | 1 (3.7) | 1 |
| **Viral infection** | 1 (7.1) | 1 | 0 | 0 | 0 | 0 | 0 | 0 | 1 (3.7) | 1 | 0 | 0 | 1 (3.7) | 1 |
| **Metabolism and nutrition disorders** | 0 | 0 | 0 | 0 | 1 (7.7) | 1 | 0 | 0 | 1 (3.7) | 1 | 0 | 0 | 1 (3.7) | 1 |
| **Dehydration** | 0 | 0 | 0 | 0 | 1 (7.7) | 1 | 0 | 0 | 1 (3.7) | 1 | 0 | 0 | 1 (3.7) | 1 |
| **Neoplasms benign, malignant and unspecified (including cysts and polyps)** | 0 | 0 | 0 | 0 | 0 | 0 | 1 (7.7) | 1 | 0 | 0 | 1 (3.8) | 1 | 1 (3.7) | 1 |
| **Breast cancer** | 0 | 0 | 0 | 0 | 0 | 0 | 1 (7.7) | 1 | 0 | 0 | 1 (3.8) | 1 | 1 (3.7) | 1 |
| **Respiratory, thoracic and mediastinal disorders** | 0 | 0 | 0 | 0 | 0 | 0 | 1 (7.7) | 1 | 0 | 0 | 1 (3.8) | 1 | 1 (3.7) | 1 |
| **Interstitial lung disease** | 0 | 0 | 0 | 0 | 0 | 0 | 1 (7.7) | 1 | 0 | 0 | 1 (3.8) | 1 | 1 (3.7) | 1 |
| **Any study treatment-related serious TEAE** | 0 | 0 | 0 | 0 | 0 | 0 | 0 | 0 | 0 | 0 | 0 | 0 | 0 | 0 |

In period 1, patients were assigned to 16 weeks of IgPro20 (0.5 g/kg/week) or IgPro10 (2 g/kg/4 weeks split over 2–5 days). Patients then received the alternative treatment during period 2. E, number of events; IgPro10, 10% intravenous human immunoglobulin, Privigen, CSL Behring; IgPro20, 20% subcutaneous human immunoglobulin, Hizentra, CSL Behring; N/n, number of patients; SAE, serious adverse events; SAF, safety analysis set; TEAE, treatment-emergent adverse events.

**Supplementary Table S2.** All recorded TEAEs, outcomes, detailed ISRs and nervous system disorders.

|  | **Period 1** | | | | **Period 2** | | | | **IgPro10  Periods (N=27)** | | **IgPro20  Periods (N=26)** | | **Total (N=27)** | |
| --- | --- | --- | --- | --- | --- | --- | --- | --- | --- | --- | --- | --- | --- | --- |
|  | **IgPro10**  **(N=14)** | | **IgPro20 (N=13)** | | **IgPro10 (N=13)** | | **IgPro20**  **(N=13)** | |  |  |  |  |  |  |
|  | **n (%)** | **E** | **n (%)** | **E** | **n (%)** | **E** | **n (%)** | **E** | **n (%)** | **E** | **n (%)** | **E** | **n (%)** | **E** |
| **All TEAEs** | | | | | | | | | | | | | | |
| **Any TEAE** | 8 (57.1) | 45 | 9 (69.2) | 18 | 5 (38.5) | 13 | 9 (69.2) | 31 | 13 (48.1) | 58 | 18 (69.2) | 49 | 22 (81.5) | 107 |
| **Serious TEAE** | 1 (7.1) | 1 | 2 (15.4) | 2 | 1 (7.7) | 3 | 3 (23.1) | 4 | 2 (7.4) | 4 | 5 (19.2) | 6 | 6 (22.2) | 10 |
| **TEAE resulting in death** | 0 | 0 | 0 | 0 | 0 | 0 | 0 | 0 | 0 | 0 | 0 | 0 | 0 | 0 |
| **TEAE leading to  discontinuation  of study treatment** | 0 | 0 | 0 | 0 | 0 | 0 | 1 (7.7) | 1 | 0 | 0 | 1 (3.8) | 1 | 1 (3.7) | 1 |
| **Outcome of TEAE** | | | | | | | | | | | | | | |
| **Recovered/Resolved** | 8 (57.1) | 40 | 9 (69.2) | 17 | 4 (30.8) | 8 | 8 (61.5) | 27 | 12 (44.4) | 48 | 17 (65.4) | 44 | 21 (77.8) | 92 |
| **Not Recovered/Not Resolved** | 3 (21.4) | 5 | 1 (7.7) | 1 | 2 (15.4) | 5 | 4 (30.8) | 4 | 5 (18.5) | 10 | 5 (19.2) | 5 | 8 (29.6) | 15 |
| **Detailed ISRs** | | | | | | | | | | | | | | |
| **Infusion site pain** | 0 | 0 | 1 (7.7) | 1 | 0 | 0 | 1 (7.7) | 2 | 0 | 0 | 2 (7.7) | 3 | 2 (7.4) | 3 |
| **Infusion site swelling** | 0 | 0 | 0 | 0 | 0 | 0 | 2 (15.4) | 3 | 0 | 0 | 2 (7.7) | 3 | 2 (7.4) | 3 |
| **Infusion site discharge** | 0 | 0 | 0 | 0 | 0 | 0 | 1 (7.7) | 1 | 0 | 0 | 1 (3.8) | 1 | 1 (3.7) | 1 |
| **Infusion site erosion** | 0 | 0 | 0 | 0 | 0 | 0 | 1 (7.7) | 1 | 0 | 0 | 1 (3.8) | 1 | 1 (3.7) | 1 |
| **Infusion site erythema** | 0 | 0 | 0 | 0 | 0 | 0 | 1 (7.7) | 1 | 0 | 0 | 1 (3.8) | 1 | 1 (3.7) | 1 |
| **Infusion site haemorrhage** | 0 | 0 | 0 | 0 | 0 | 0 | 1 (7.7) | 1 | 0 | 0 | 1 (3.8) | 1 | 1 (3.7) | 1 |
| **Infusion site reaction** | 0 | 0 | 0 | 0 | 0 | 0 | 1 (7.7) | 1 | 0 | 0 | 1 (3.8) | 1 | 1 (3.7) | 1 |
| **Infusion site vesicles** | 0 | 0 | 0 | 0 | 0 | 0 | 1 (7.7) | 1 | 0 | 0 | 1 (3.8) | 1 | 1 (3.7) | 1 |
| **Injection site hypersensitivity** | 0 | 0 | 1 (7.7) | 1 | 0 | 0 | 0 | 0 | 0 | 0 | 1 (3.8) | 1 | 1 (3.7) | 1 |
| **Injection site mass** | 0 | 0 | 0 | 0 | 0 | 0 | 1 (7.7) | 1 | 0 | 0 | 1 (3.8) | 1 | 1 (3.7) | 1 |
| **Thromboembolic events** | 0 | 0 | 0 | 0 | 0 | 0 | 1 (7.7) | 1 | 0 | 0 | 1 (3.8) | 1 | 1 (3.7) | 1 |
| **Detailed nervous system disorders** | | | | | | | | | | | | | | |
| **Headache** | 5 (35.7) | 11 | 1 (7.7) | 1 | 0 | 0 | 0 | 0 | 5 (18.5) | 11 | 1 (3.8) | 1 | 6 (22.2) | 12 |
| **Dizziness** | 1 (7.1) | 2 | 0 | 0 | 0 | 0 | 1 (7.7) | 2 | 1 (3.7) | 1 | 1 (3.8) | 2 | 1 (3.7) | 4 |
| **Somnolence** | 0 | 0 | 1 (7.7) | 1 | 0 | 0 | 0 | 0 | 0 | 0 | 1 (3.8) | 1 | 1 (3.7) | 1 |
| **Sciatica** | 0 | 0 | 1 (7.7) | 1 | 0 | 0 | 0 | 0 | 0 | 0 | 1 (3.8) | 1 | 1 (3.7) | 1 |
| **Anosmia** | 0 | 0 | 1 (7.7) | 1 | 0 | 0 | 0 | 0 | 0 | 0 | 1 (3.8) | 1 | 1 (3.7) | 1 |

In period 1, patients were assigned to 16 weeks of IgPro20 (0.5 g/kg/week) or IgPro10 (2 g/kg/4 weeks split over 2–5 days). Patients then received the alternative treatment during period 2. E, number of events; IgPro10, 10% intravenous human immunoglobulin, Privigen, CSL Behring; IgPro20, 20% subcutaneous human immunoglobulin, Hizentra, CSL Behring; ISR, infusion site reaction; N/n, number of patients; TEAE, treatment-emergent adverse events.

**Supplementary Table S3**. mRSS total score and mRSS responders by sequence and treatment.

|  | **Sequence A  (IgPro20/IgPro10, N=13)** | **Sequence B  (IgPro10/IgPro20, N=14)** | **IgPro10 (N=27)** | **IgPro20 (N=27)** | **Total (N=27)** |
| --- | --- | --- | --- | --- | --- |
| **mRSS total score**^†^ | | | | | |
| Baseline, mean (SD) | 23.8 (5.8) | 25.0 (7.1) |  |  | **24.4 (6.4)** |
| **Change from Baseline** | | | | | |
| Week 17 before infusion, mean (SD) | -5.7 (4.8) | -3.4 (4.3) |  |  | **-4.6 (4.6)** |
| Week 32 end of treatment, mean (SD) | -7.6 (6.3) | -7.5 (5.4) |  |  | **-7.6 (5.8)** |
| **mRSS responders*** | | | | | |
| **Week 1 to Week 17** | | | | | |
| n (%) | 6 (46.2) | 5 (35.7) |  |  | **11 (40.7)** |
| 95% CI for the proportion | 23.2, 70.9 | 16.3, 61.2 |  |  | **24.5, 59.3** |
| **Week 17 to Week 32** | | | | | |
| n (%) | 2 (15.4) | 2 (14.3) |  |  | **4 (14.8)** |
| 95% CI for the proportion | 4.3, 42.2 | 4.0, 39.9 |  |  | **5.9, 32.5** |
| **Week 1 to Week 32** | | | | | |
| n (%) | 9 (69.2) | 9 (64.3) |  |  | **18 (66.7)** |
| 95% CI for the proportion | 42.4, 87.3 | 38.8, 83.7 |  |  | **47.8, 81.4** |
| **During assessment period^§^** | | | | | |
| n (%) |  |  | 7 (25.9) | 8 (29.6) |  |
| 95% CI for the Proportion |  |  | 13.2, 44.7 | 15.9, 48.5 |  |

*Response criterion: change from reference visit ≤ -5 and percent change from reference visit ≤ -25%.

^†^mRSS total score ranges from 0 to 51 (higher is worse, negative change is improvement)

^§^Assessment period for IgPro10 = Week 1 to Week 17 (Sequence B) and Week 17 to Week 32 (Sequence A); for IgPro20 = Week 1 to Week 17 (Sequence A) and Week 17 to Week 32 (Sequence B). Percentages are calculated with the number of subjects in each sequence/ treatment as the denominator (N).

N, number of subjects; n, number of subjects meeting criterion; CI, Wilson score confidence interval; mRSS, modified Rodnan skin score.
